# MRI assessment of treatment response

**DOI:** 10.1186/1470-7330-15-S1-O26

**Published:** 2015-10-02

**Authors:** Ann D King

**Affiliations:** 1 Chinese University of Hong Kong, China

## 

Locoregional failure occurs in approximately 25-50% of patients with head and neck squamous cell carcinoma (HNSCC), who are treated with chemoradiotherapy (CRT). Timely identification of treatment failure allows salvage surgery to be undertaken. Imaging the post-treatment neck therefore has an important role for the early identification of a residual/recurrent tumours while also preventing unnecessary biopsies or surgery in patients with expected post-treatment changes. This lecture will concentrate on the post CRT evaluation of primary and nodal sites in patients with HNSCC, using morphological assessment by MRI.

MRI assessment of the post treatment primary tumour bed is based on CT criteria and can be divided into (1) expected post treatment changes (low risk); (2) focal mass < 1cm or asymmetry (indeterminate risk); (3) focal mass ≥ 1cm (high risk). In addition MRI signal intensity can provide further information. Residual/recurrent tumours tend to have the same signal intensity as untreated tumours on all sequences, and in this regard the T2 weighted images are valuable in distinguishing tumour (intermediate signal intensity) from scar tissue (low signal intensity) and inflammation (high signal intensity). However, there may still be some overlap in the appearance of post treatment change and tumour.

The assessment of nodal response can be even more problematic, especially in the early post-treatment period. Reported morphological criteria for identifying nodal treatment response vary but most frequently nodal control is based on size (<1- 1.5cm); % size reduction (>75%-90%); absence of focal abnormalities such as necrosis; absence of extranodal neoplastic spread (ENS). These criteria are often combined and report high NPVs, valuable for excluding residual malignant nodes, but the PPVs are low. The poor diagnostic performance of these morphologic criteria is partly because necrosis and ENS are inaccurate signs for residual nodal cancer, and their presence reduces the accuracy of size measurements. Of note necrotic sterile nodes often take longer to decrease in size than solid sterile nodes.

Interactive clinical cases of primary and nodal HNSCC will be used to illustrate the expected post treatment findings, residual/recurrent tumours, and indeterminate findings that cause a diagnostic dilemma. DWI will also be illustrated and there will be a brief comparison of MRI and FDG PET-CT.
